# Occipital repetitive transcranial magnetic stimulation does not affect multifocal visual evoked potentials

**DOI:** 10.1186/s12868-020-00600-5

**Published:** 2020-11-23

**Authors:** Robert Kolbe, Aykut Aytulun, Ann-Kristin Müller, Marius Ringelstein, Orhan Aktas, Alfons Schnitzler, Hans-Peter Hartung, Stefan Jun Groiss, Philipp Albrecht

**Affiliations:** 1 Department of Neurology, Medical Faculty, Heinrich-Heine University, Moorenstraße 5, 40225 Düsseldorf, Germany; 2 Institute of Clinical Neuroscience and Medical Psychology, Medical Faculty, Heinrich-Heine University, Düsseldorf, Germany

**Keywords:** Multifocal visual evoked potentials, Repetitive transcranial magnetic stimulation, Occipital stimulation, Excitatory stimulation, Long term potentiation, Cortical plasticity

## Abstract

**Background:**

To identify mechanisms of cortical plasticity of the visual cortex and to quantify their significance, sensitive parameters are warranted. In this context, multifocal visual evoked potentials (mfVEPs) can make a valuable contribution as they are not associated with cancellation artifacts and include also the peripheral visual field.

**Objective:**

To investigate if occipital repetitive transcranial magnetic stimulation (rTMS) can induce mfVEP changes.

**Methods:**

18 healthy participants were included in a single-blind crossover-study receiving sessions of excitatory, occipital 10 Hz rTMS and sham stimulation. MfVEP was performed before and after each rTMS session and changes in amplitude and latency between both sessions were compared using generalized estimation equation models.

**Results:**

There was no significant difference in amplitude or latency between verum and sham group.

**Conclusion:**

We conclude that occipital 10 Hz rTMS has no effect on mfVEP measures, which is in line with previous studies using full field VEP.

## Background

The procedure of repetitive transcranial magnetic stimulation (rTMS) uses a strong magnetic field generated by a coil placed on the skull to induce an electric current in the upper layer of the cortex [1]. While single magnetic field pulses can be used to stimulate the cortical area, repetitive pulses can modify the excitability in an excitatory or inhibitory manner. In general, higher frequencies than 5 Hz [2] have a facilitating effect while lower frequencies around 1 Hz are considered inhibitory [3].

The direct effects of the rTMS depend on the location, intensity and frequency of stimulation. At the visual cortex, they manifest themselves in the form of short-lived light sensations called phosphenes [4]. Earlier investigations have demonstrated that these visual sensations originate from the terminal parts of the optic radiation close to its ending in V1 as well as from tracts leading back from V2 and V3 to V1, so that a major role of V1 can be assumed [5]. An overview study reviewing recent findings about the effects of rTMS on a neurobiological level found evidence for changes in the expression patterns of several target proteins after excitatory and inhibitory rTMS [6]. For the visual cortex, a previous study reported that 1 Hz stimulation leads to decreased amplitudes in full-field visual evoked potentials (ffVEP) recorded directly after rTMS [7]while excitatory protocols with 10 Hz rTMS stimulation had no effects on the ffVEP [7]. Another study investigating the effect of excitatory rTMS on habituation to ffVEP could not find any effect in healthy subjects as well [8].

Visual evoked potentials (VEP) are a neurophysiological examination technique of the visual system that is conducted by recording the amplitude and latency of a signal evoked by exposure to a visual stimulus usually consisting of a reversing checkerboard pattern. For the recording of the cortical activity electrodes are placed on the skull of the proband over the occipital cortex. The visual stimulation and the recording of cortical activity can be performed simultaneously on the whole visual field or separately on separate regions of the visual field resulting in full-field visual evoked potentials (ffVEP) or multifocal visual evoked potentials (mfVEP).

The aim of this study was to investigate whether excitatory rTMS of the visual cortex results in amplitude and/or latency changes of multifocal visual evoked potentials (mfVEP), which are more sensitive for change compared to ffVEP. Such changes could constitute a sensitive and objective neurophysiological correlate for the cortical plasticity of the visual cortex. This could be of great value to investigate adaptive or compensatory mechanisms in pathological conditions involving the afferent visual pathway such as optic neuritis. FfVEPs are prone to cancellation artifacts and therefore mainly represent the central lower part of the visual field. In contrast, mfVEPs allow for a more precise investigation of the visual system covering a large part of the visual field (24°of excentricity) [9]. This area is divided into separate regions leading to a higher spatial resolution than ffVEP. For each of these regions a separate amplitude and latency is detected with an accuracy in the nV-range, which is far superior to ffVEPs measures in µV. Therefore, the mfVEP may be a more sensitive instrument to reevaluate the long-term-effects of excitatory rTMS which did not lead to significant changes in ffVEP [7, 8]. Former investigations have shown that the signals recorded by mfVEP are largely generated in V1, which is consistent with our location of stimulation [10].

## Materials and methods

### Participants

18 healthy participants (8 male, 10 female) were included in the study. The age of the participants ranged from 18 to 61 years with a mean of 30.13 ± 11.83 (standard deviation) years. No participant aborted participation during the study.

Exclusion criteria were implanted electronic devices or other metal objects, pregnancy, a history of epileptic seizure(s) or taking medication lowering the seizure threshold as well as a history of any neurological or ophthalmological disorder.

### mfVEP

**Fig. 1:**
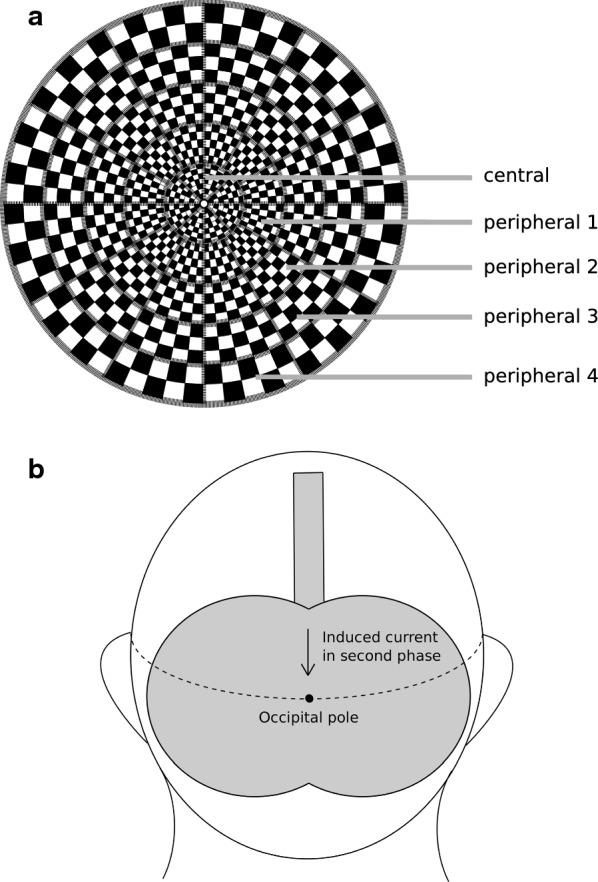
Mode of stimulation. **a** Schematic visualization of the sectioning of the examined part of the visual field into different regions with an increasing level of eccentricity. **b** Illustration of the location of stimulation used in the verum group. In every subject, the butterfly coil was positioned above the inion in the depicted angle

Every participant was invited to four sessions of mfVEP recordings performed immediately before and after rTMS and immediately before and after sham stimulation. mfVEP assessments were performed with a VISIONSEARCH1 mfVEP system using the TERRA software as previously described [11, 12]. In brief, simultaneous multi-focal stimulation of 56 segments of the visual field (24 of eccentricity) was performed via a 68 s pseudorandom sequence (Fig. 1a) using a reversing checkerboard pattern. The visual response was recorded with 2-channels by electrodes glued to the scull with collodion at previously defined positions of a cross around the inion in accordance with prior investigations using mfVEP [11, 12]. Amplitude and latency were recorded from a horizontal and a vertical channel for each segment and the stimulation was repeated until the noise was reduced below 10% of the recorded traces or a maximum of 12 runs was attained. The channel with the best amplitude was used for analysis.

### rTMS

All participants were assigned to a session of verum occipital and a session of sham frontal stimulation without being aware about which was the control session. The two sessions were applied with an intersession interval of at least 24 h to exclude carry over effects. Biphasic rTMS was performed using a MC-B70 butterfly coil connected to a DANTEC MagLite magnetic stimulator.

The verum rTMS was conducted at the inion with downward current induced in the brain in the second phase (Fig. 1b). The phosphene threshold was identified by stimulation with single pulses at the inion using a modified relative frequency method [13] with the electrodes recording the mfVEP attached to the skull. To this end, the stimulator output was initially set to 35% and increased in intervals of 5% until a visual sensation was perceived. Prior to testing, the participants were instructed to announce the appearance of any of the following visual symptoms: a short flash of light or a briefly appearing structure resembling a line or a cloud. rTMS was administered in a dimmed room. Using this protocol, phosphenes could be elicited in all participants at stimulation intensities of 35–65%. After dertermining the phosphene threshold, 18 cycles of excitatory rTMS with a duration of 5 s and a frequency of 10 Hz were applied in 10-s intervals at the phosphene threshold.

The sham rTMS was conducted at the frontal cortex in a midsagittal plane, halfway between nasion and vertex as stimulation of the frontal cortex with low stimulation intensity is unlikely to affect mfVEP recordings. The field strength of the stimulation was set to 35% of the maximally possible stimulator output and stimulation protocol was identical to the verum rTMS to generate a sensation in the participants similar to the verum rTMS.

### Statistical evaluation

Statistical analyses were performed using Microsoft Excel, GraphPad Prism 5.00 and IBM SPSS Statistics 20.0.0.2. The differences in amplitude and latency between pre- and post-stimulation mfVEPs were calculated for both sessions. The differences of amplitude and latency for each stimulation condition were compared in general and for every eccentricity using a GEE-model correcting for within subject inter-eye correlations. A combined Z-score consisting of the increase in amplitude and the decrease in latency was also calculated for both stimulation conditions and compared by GEE-analysis.

A Bonferroni correction was performed to correct for multiple testing. Adjusting the initial P-value of 0.05 for 18 tests led to a corrected P-value of 0.0028, so P-values below 0.28% (p < 0.0028) were considered significant.

## Results

### Effects of rTMS on amplitude

In general, we did not detect any effect of rTMS on mfVEP amplitudes (GEE). The mean amplitude decreased by 2.54 nV after active rTMS, while we observed an increase of amplitude by 4.63 nV after sham rTMS leading to a mean difference of 7.17 nV between both stimulation conditions, which was not considered significant (p = 0.155).

**Fig. 2:**
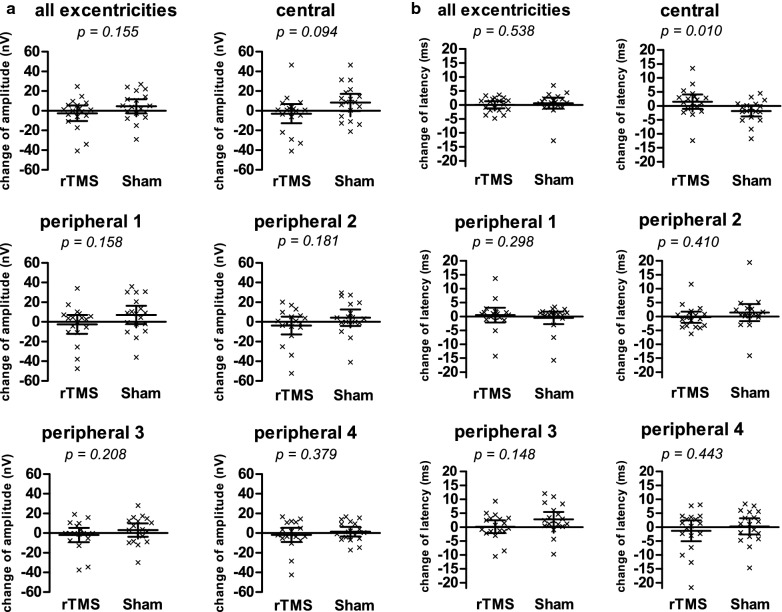
Effects of verum and sham stimulation on amplitude and latency. **a** Scatter diagram showing the difference in amplitude between the mfVEP examinations before and after stimulation for rTMS group and sham group. Statistical evaluation has been performed in general and for each eccentricity separately. Each dot represents one subject. In every diagram, the arithmetic mean of the amplitude is marked by a horizontal line with whiskers indicating the 95% confidence interval. **b** Scatter diagram showing the difference in latency between the mfVEP examinations before and after stimulation for rTMS group and sham group. Statistical evaluation has been performed in general and for each eccentricity separately. Each dot represents one subject. In every diagram, the arithmetic mean of the latency is marked by a horizontal line with whiskers indicating the 95% confidence interval

Analyzing all eccentricities separately also revealed no significant effect of rTMS on amplitudes (results presented in Table 1 and Fig. 2a).

**Table 1:** Difference between verum and sham stimulation in amplitude, latency and a combined z-Score

	Change in amplitude (nV)			Change in latency (ms)			Change in Z-score		
	Verum	Sham	p-value	Verum	Sham	p-value	Verum	Sham	p-value
Full field	− 2.54	+ 4.63	0.155	+ 0.01	+ 0.60	0.538	− 0.079	+ 0.063	0.528
Central	− 2.92	+ 8.47	0.094	+ 1.49	− 1.84	0.010	− 0.282	+ 0.496	0.006
Peripheral 1	− 2.49	+ 7.01	0.158	+ 0.50	− 0.47	0.298	− 0.140	+ 0.275	0.121
Peripheral 2	− 3.63	+ 4.24	0.181	− 0.24	+ 1.40	0.410	− 0.079	− 0.053	0.941
Peripheral 3	− 2.00	+ 3.20	0.208	+ 0.15	+ 2.83	0.148	− 0.081	− 0.269	0.527
Peripheral 4	− 1.80	+ 1.51	0.379	− 1.33	+ 0.26	0.443	+ 0.118	+ 0.013	0.744

### Effects of rTMS on latency

We did not observe an effect of rTMS on mean peak1 latency (GEE). The mean latency remained almost constant with an increase of 0.01 ms and 0.6 ms after rTMSfor the verum and sham condition, respectively (p = 0.982 and p = 0.512). The latency change did not differ between verum and sham stimulation (p = 0.538, GEE).

Furthermore, we observed no effect of rTMS on latency in separate analyses of the different eccentricities (see Table 1, Fig. 2b), except for the central field, which showed a tendency for an increase of latency by 1.49 ms after verum stimulation and a decrease by 1.84 ms after shamrTMS (p = 0.215 and p = 0.044, respectively). This difference of 3.33 ms between both stimulation conditions was not considered significant (p = 0.010 > 0.0028).

### Effects of rTMS on a combined Z-score consisting of the increase in amplitude and the decrease in latency

To increase the sensitivity for a parallel deterioration of amplitudes and latencies in participants we calculated a combined Z-score representing an increase in amplitude and a decrease in latency. However, we observed no effect of rTMS on the combined Z-score, which decreased by 0.079 and 0.063 after verum and sham rTMS, respectively (verum vs sham p = 0.528).

Analyzing the combined Z-scores separately for the different eccentricities revealed no differences between both stimulation conditions (see Table 1), except for the central field, where we observed a tendency to an increase of the combined Z-score after sham rTMS of 0.496, while the Z-score after verum rTMS decreased by 0.282 resulting in a difference of 0.778, which was not significant (p = 0.006 > 0.0028).

Table showing the increase of amplitude (nV), latency (ms) and a combined Z-score between the mfVEP measurements before and after stimulation. The Z-score consists of the increase in amplitude and the decrease in latency. The differences between both measurements are shown for verum and sham stimulation in general and for each eccentricity separately. The depicted p-values have been determined using a GEE-model correcting for within subject inter-eye correlations. P-values below 0.0028 were considered significant.

## Discussion

In line with previous results using ffVEP technology [7], we observed no significant effects of a 10 Hz rTMS stimulation of the occipital cortex on amplitude or latency of visual evoked potentials [7]. The fact that this finding initially made with ffVEP-recordings could be confirmed with the more sensitive mfVEP methodology indicates that the lack of rTMS response does not seem to be a sensitivity issue of ffVEPs and that it also applies for the more peripheral parts of the visual field not assessed by ffVEP. We observed a tendency towards a decrease in latency after sham stimulation in the central field. To the best of our knowledge, there are no reports of an influence on the visual system by rTMS of the frontal cortex in a mid-sagittal plane, which we therefore consider a statistical artifact. A possible reason for this observation could be learning effects linked to the study protocol. We have to acknowledge that verum stimulation was done before sham in all participants so we cannot completely exclude order effects, which has to be mentioned as a limitation of our study. However, previous investigations have shown a very good test–retest reliability of the mfVEP assessments [14]. Therefore, we believe that repeating the mfVEP assessment is unlikely to result in latency decrease or amplitude increase at the second measurement and have to point out that we observed no significant differences.

## Conclusions

In summary, we conclude that excitatory 10 Hz rTMS of the occipital cortex had no effect on mfVEP outcomes in healthy controls, in our study. This suggests that mfVEP, despite its high sensitivity, may not be suited to investigate cortical plasticity of the visual cortex in healthy conditions, which is in line with the results reported for ffVEP. The question if cortical plasticity may increase in the context of anterior visual pathway damage like optic neuritis and could then be elicited by rTMS and VEP remains subject to speculation. For such studies higher numbers of participants and other rTMS protocols, e.g. utilizing fMRI to validate the correct location of stimulation over V1, should be considered.

## Data Availability

The datasets used and/or analysed during the current study are available from the corresponding author on reasonable request.

## Abbreviations

ffVEP: Full-field visual evoked potentials
GEE: General estimating equation
mfVEP: Multifocal visual evoked potentials
rTMS: Repetitive transcranial magnetic stimulation
